# Staff Perspectives on a Tablet-Based Intervention to Increase HIV Testing in a High Volume, Urban Emergency Department

**DOI:** 10.3389/fpubh.2017.00170

**Published:** 2017-07-11

**Authors:** Ian David Aronson, Honoria Guarino, Alexander S. Bennett, Lisa A. Marsch, Marya Gwadz, Charles M. Cleland, Laura Damschroder, Theodore C. Bania

**Affiliations:** ^1^National Development and Research Institutes, New York, NY, United States; ^2^Center for Technology and Behavioral Health, Geisel School of Medicine at Dartmouth, Lebanon, NH, United States; ^3^Center for Drug Use and HIV Research (CDUHR), New York University College of Nursing, New York, NY, United States; ^4^Implementation Pathways, Ann Arbor, MI, United States; ^5^Icahn School of Medicine at Mount Sinai, Mount Sinai St. Luke’s, Mount Sinai West, New York, NY, United States

**Keywords:** HIV, video, emergency medicine, tablet computers, implementation science

## Abstract

Emergency departments (EDs) frequently serve people who have limited, if any, additional interactions with health care, yet many ED patients are not offered HIV testing, and those who are frequently decline. ED staff (*n* = 13) at a high volume urban ED (technicians, nurses, physicians, and administrators) were interviewed to elicit their perspectives on the feasibility and acceptability of a tablet-based intervention designed to increase HIV test rates among patients who initially decline testing. Content-based thematic analysis of semi-structured interviews indicated overall support for interventions to increase HIV testing, but a lack of available staff resources emerged as a potential barrier to widespread implementation. Also, some ED staff questioned whether it was appropriate to shift responsibility for public health services, such as HIV testing, to the ED instead of a primary care setting. Although tablet-based interventions have been shown effective in high volume ED settings and can potentially increase HIV test rates among hard-to-reach populations, additional effort is now required to better integrate this type of intervention into existing workflows.

## Introduction

Despite the ongoing problem of undiagnosed HIV, many emergency department (ED) patients are not offered testing and many of those who are offered testing decline (1, 2). Nonetheless, EDs can be key entry points within the HIV care continuum (3) because many people at increased risk for HIV infection have no interaction with the health-care system other than visits to an ED (2, 4). Because EDs house existing clinical structures that can efficiently process HIV tests and deliver results, EDs provide key opportunities for diagnosis and then linkage to care for patients who test positive (3), along with interventions aimed at reducing risk for those who test negative.

In 2006, the Centers for Disease Control and Prevention recommended routine HIV testing for all ED patients, with limited exceptions (5), and as of 2013 the US Preventive Services Task Force required public and private insurance to cover the costs of HIV testing for patients 15–65 years of age without patient co-payments or other out-of-pocket patient expense (6). Still, recent studies indicate poor uptake of testing and thus significant missed opportunities to identify undiagnosed HIV infection (1, 2).

For example, a recent study by Hsieh and colleagues found that almost two-thirds of patients in a Baltimore ED were not offered HIV testing, and among those offered testing, more than half declined. They also found that prevalence of undiagnosed HIV was three times higher in those who were not offered testing compared to those who were offered testing (1). These findings echo results from a study conducted by Czarnogorski and colleagues who found that patients who declined HIV testing in a Washington, DC area ED were 2.74 times more likely to have undiagnosed HIV compared to patients who accepted HIV testing (7).

Zuckeri and colleagues studied HIV screening rates at a Newark, NJ ED, where established programs provide HIV counseling, testing, and referral services seven days a week (2). The study found that, among a sample of 27,312 eligible visits from October 2014 through February 2015, only 9% of eligible patients were screened for HIV (2). Zucker and colleagues describe this as particularly worrisome, because patients in high-prevalence communities who have no other access to care may have expected to be screened when they visited the ED or may falsely assume they have been tested for HIV during an ED visit, even when they have not (2).

New York State law requires health-care staff in EDs and other settings to offer HIV testing to all patients aged 13–64, with limited exceptions, yet far more patients decline HIV testing compared to those who accept (8). In addition, many patients are not offered testing if they cannot provide informed consent, are critically ill, or are outside the age range of 13–64 years. Hsieh and colleagues note that some facilities may lack adequate personnel to approach all patients eligible for HIV testing and suggest that an integrated, streamlined, automated approach may help address barriers to offering HIV tests (1).

Our research team developed a series of brief tablet-based interventions that align with the streamlined, automated approach suggested by Hsieh and colleagues, to increase HIV testing by targeting patients who initially decline tests offered by hospital staff. Participants in our studies used handheld computers to complete an automated HIV risk assessment, pre–post HIV knowledge assessments, and watch brief videos about HIV prevention and testing. We have piloted the interventions within an exceptionally high volume New York City ED (8–10). In each of our intervention pilots, roughly half of patients approached agreed to participate, and approximately one-third of participants who initially declined HIV tests offered by ED staff at triage, accepted an HIV test after completing the intervention.

However, in these pilot studies, patients were recruited by volunteer research assistants. In order to understand how such an intervention may be implemented on a larger scale as routine practice in busy EDs, we must first gain a better understanding of who should deliver the intervention (e.g., physician and nurse) and at what point in a patient’s visit the intervention should be offered to maximize effectiveness and prevent longer stays in the ED. Perhaps most importantly, we need to examine the best process for reaching the greatest number of individuals who are most in need of testing. This is especially challenging in light of evidence that populations with the greatest HIV risk and the highest rates of undiagnosed HIV may be the least likely to accept testing when offered.

To date, ED staff perspectives on the implementation of technology-based interventions to increase HIV testing have been underexplored. To develop a more thorough understanding of staff perspectives on one such intervention, which we believe is necessary before these tools can be widely implemented, our research team conducted a Hybrid Type 1 Effectiveness-Implementation trial with the dual aims of evaluating clinical outcomes while integrating a process evaluation to generate knowledge to inform further implementations (11). We recruited a sample of 300 patients in a high volume New York City ED, all of whom had declined HIV testing offered by hospital staff, to complete a brief tablet computer-based intervention (mean time to completion was 8 min). At the end of the intervention 91 participants (30.33%), accepted an HIV test. We have described our interventions’ preliminary effectiveness, as well as feasibility and acceptability from patients’ point of view, in previous publications [e.g., Ref. (8, 12)]. The current paper aims to explore ED staff perspectives on implementation of our most recent tablet-based intervention and to elicit potential barriers to implementation.

## Materials and Methods

The objective of the current study was to examine ED staff perspectives on key issues regarding the implementation of the tablet intervention, including who should deliver the intervention if it were adopted as standard practice and how the intervention might be best integrated into existing workflows, as well as general staff perspectives on routine HIV testing in EDs. Semi-structured interviews were conducted with ED staff shortly after the most recent trial was completed. All interviewees worked in the ED where the trial was conducted.

### Participants

Emergency department staff (*n* = 13) were purposively sampled to represent the range of roles and levels of responsibility within the ED where the study was conducted and to include staff with direct knowledge of the intervention. Two of the authors with access to staff rosters (Theodore C. Bania and Ian David Aronson) decided which ED staff to approach for interviews. The sampling strategy was designed to include maximum variation (13) in role within the ED (e.g., administrator, physician, nurse, and technician) and to select individuals who were both knowledgeable about HIV testing in the ED and willing to discuss their opinions. Participants provided written informed consent to be interviewed. At the end of each interview, participants received a $25 gift card.

As presented in Table 1, the sample included six nurses, three physicians, two medical testing technicians, and two administrators. Eight interviewees were female, and the sample was racially diverse (Asian, African American, White, and Latino).

**Table 1:** Profession, age, race, and gender of interviewees.

Profession	Age	Race	Gender
Physician	Mid 60s	White	Male
Nurse	Mid 30s	Asian	Female
Administrator	Early 60s	White	Male
Administrator	Late 40s	White	Male
Technician	Mid 20s	Black	Female
Technician	Early 30s	Black	Female
Nurse	Early 60s	Black	Female
Nurse	Mid 40s	Black	Female
Nurse	Mid 50s	Asian	Female
Nurse	Late 40s	Asian	Female
Physician	Early 40s	Asian	Female
Physician	Mid 30s	White	Male
Nurse	Mid 20s	Latino	Male

### Interview Approach and Materials

Semi-structured interviews were guided by a flexible interview protocol consisting of a defined set of topics followed by open-ended questions and optional probes designed to encourage interviewees to voice their own perspectives. Interviews began with general questions regarding the staff member’s experiences with and views of HIV screening in the ED and potential strategies for increasing the number of patients who agree to be tested and proceeded to more specific queries such as: how to implement a tablet intervention without interfering with staff workflow; when in the course of a patient’s visit this type of intervention should be offered; which staff are best positioned to deliver such an intervention; salient barriers to integrating a tablet intervention as part of routine practice in the ED; and how these barriers could be addressed.

To elicit potential barriers, the interview guide was informed by the Consolidated Framework for Implementation Research (CFIR) (14). The CFIR describes constructs that have been found to influence implementation success including factors related to intervention characteristics (e.g., complexity), outer setting (e.g., reimbursement policy), inner setting (e.g., available resources), individual characteristics (e.g., self-efficacy to use the intervention), and process (e.g., planning).

All interviews were conducted by the first author in a private space within the ED. Interviews were digitally audio-recorded and lasted approximately 20 min. In light of the faced-paced ED setting, interviews were designed to be brief and tightly focused to avoid undue burden on staff. The interview guide and consent forms were approved by Institutional Review Boards at the hospital site and the study team’s home institution, NDRI.

### Data Analysis

Two experienced qualitative researchers (Alexander S. Bennett and Honoria Guarino) conducted content-based thematic analysis of verbatim interview transcripts in the software program *MAXQDA* (VERBI Software, Berlin, Germany). Focused on the manifest content of interviewees’ speech (15), this approach is well-suited to the study’s objective to better understand staff members’ consciously held views regarding the implementation of a tablet-based intervention in the ED. The analysis followed a hybrid deductive–inductive process in which the analysts coded transcripts for themes directly related to the research aims while also attending to emergent, previously unanticipated themes. After an initial review of the transcripts, the analysts collaboratively developed a preliminary codebook that was refined and elaborated through subsequent review of transcripts; criteria for assigning codes to text segments were developed and emergent themes were added, as necessary. Using the final codebook, each analyst independently coded all transcripts. The few discrepancies between assigned codes were resolved by consensus. The study team then used coding reports to form an overarching interpretation by organizing discrete themes into four higher-order categories of topically related themes; this interpretation provides an organizing framework for the following presentation of study results. Unless otherwise indicated, the quoted interview segments represent dominant themes expressed by multiple interviewees.

## Results

Salient themes expressed by staff clustered around three issues relevant to the successful implementation of a tablet intervention to increase HIV testing in the ED: the specific people best suited to deliver the intervention; the stage during a patient’s ED visit that is optimal for delivering the intervention, and strategies staff view as critical to facilitating uptake of HIV screening in the ED. In addition, a fourth category of themes, relating staff’s perspectives regarding two challenges associated with universal HIV screening in the ED that were not specifically anticipated but emerged in staff interviews, is described.

### Who Should Deliver a Tablet Intervention?

An obvious question that emerges from our research is who should administer the intervention if it were adopted as routine practice. Although our team’s tablet interventions are designed to be largely self-directed once in the hands of a patient, human contact, and coordination are required because someone must approach each patient to introduce the intervention, briefly explain its purpose, and give a tablet computer to those who agree to participate. Interviewees offered varying opinions about who should do this.

Some interviewees, including nurses, suggested that nurses would be the best candidates to take on this task. As one nurse explained, HIV testing is a logical expansion of their care-taking role in the ED:

Most likely, it’s probably going to have to be the nurse…I mean, the nurse is the one who’s with that patient most of the time, taking care of the patient, giving the patient medicines and such. …I can’t see the doctor doing it. I think [the patient] will be more open, too, for the nurse who’s taking care of them and also to do this with them as a teacher. (#71, nurse)

However, a number of interview responses suggest that a lack of available staff resources could present a clear barrier to implementation of a tablet-based intervention. ED staff are already very busy, and few have time to pick up additional responsibility. Several interviewees suggested that, ideally, a dedicated staff person whose sole responsibility is HIV testing would be tasked; this would maximize efficiency and provide continuity in care, as the same individual who conducts the rapid antibody testing could also deliver a related tablet intervention. In fact, staff reported that in years past, when pre- and posttest counseling was a mandated accompaniment to all HIV testing in New York State, the ED had such a position.

Given the lack of continued funding for a dedicated HIV testing position, as well as a lack of available time on the part of existing staff, some interviewees suggested that various categories of unpaid personnel, such as medical students or volunteers, would be best choices to deliver a tablet intervention. A physician expanded on this, stating, “Students would be ideal because they’re always usually looking for things to do and they want to be involved in care. So this would make them feel good too.”

In addition, several interviewees expressed the opinion that staff in various positions could potentially deliver a tablet intervention, depending on the needs and opportunities presented by a given situation. For example, a testing technician stated, “I think it could be anyone really, basically. Whoever gets to the patient first.” The staff members who endorsed this view tended to emphasize the importance of flexibility and teamwork to maintaining an effective and efficient process of intervention delivery. As a nurse explained:

Sometimes like in the triage [the patient] will say no and then when the doctor examines the patient they will tell the doctor, “Oh, I want to have [an HIV test] done.” So, I think it’s like the team, like teamwork or something. (#70, nurse)

In short, while staff frequently noted the potential importance of an intervention designed to increase HIV testing, a lack of staff resources emerged as an obvious barrier to implementation. Across roles (technician, nurse, doctor), staff we spoke with described the ED as an environment where people were already working at capacity, and there was no agreement as to who, if anyone, should actually be charged with routinely administering even a brief new intervention.

### When Should a Tablet Intervention Be Offered to ED Patients?

Staff perspectives regarding the best point during a patient’s visit to deliver a brief tablet intervention were more consistent. Most interviewees noted that this aspect of intervention implementation would be best served by an adaptive strategy in which staff members capitalize on brief lulls in a patient’s visit. When asked how to implement the tablet intervention without impeding work flows, one nurse answered:

By not making it a specific point in the treatment, [rather] by making it a downtime activity. So say the patient has to go to the X-ray but there’s no transport ready maybe that’s the time that they want to go there. Or say the patient has been checked in but there is no room available yet, that’s the time that they can go. Or say the patient has seen the doctor and they are waiting for a lab result or another type of result or an X-ray result, that’s the time that they should be able to do it by keeping it flexible. So that on the patient’s downtime, that’s when they’re offered this video so that they’re not distracted, we’re not distracted. It’s at a time when they’re waiting for something. (#72, nurse)

Despite the recognized need for flexibility, interviewees agreed that there were general times within a typical patient visit that would be most appropriate for delivering a tablet intervention and conducting HIV screening:

I think you’d want to get to them early in their visit but after they’ve had their initial assessment…once they’re triaged, once they are in a room …[once] the nurse has been in there at least to get things started, then I think it’s okay to do it. The reason I say earlier in the visit is that—you know, toward the end of the visit people have been waiting a while, maybe some of the results are in and they have mentally—“Yeah, I’d love the test but I just need to get out of here at this point.” So I think early on they’d be more accepting and because it does take 20 minutes just to run the test that you don’t want that to be the thing that’s holding them up at the end because then they’d be resistant to it or not want it. (#76, ED administrator)

These parameters were echoed by a nurse who emphasized that patients would be more receptive to an intervention after they know their primary complaint is being addressed: “People want their issues to be addressed…They want their condition seen first, taken care of, then you can move onto other things.”

### How Can EDs Facilitate Uptake of Routine HIV Testing?

With regard to strategies to maximize uptake of HIV testing in the ED, nurses and testing technicians emphasized the importance of anticipating and responding to patients’ needs and concerns when approaching ED patients about HIV screening. Adopting a non-judgmental stance was seen as critical to an effective approach in their view, as patients may feel implied judgment when providers enquire about their willingness to be tested for HIV.

In light of the stigma that continues to be associated with HIV, maintaining patients’ privacy is also paramount; many patients are reluctant or unwilling to accept an HIV test in the presence of a partner, friend or relative, fearing that agreeing to a test will lead others to assume they have engaged in risky sex- or drug-related behavior. Several frontline staff underscored the importance of broaching the topic of HIV testing privately, one-on-one:

[Y]ou should be like in a certain area, like you have some privacy. Yeah they will agree to it, if it’s—because if it’s like everybody can hear it, of course they will like double guessing or they will say, “Oh, I’m not comfortable with it,” so having a room, a single room, a private room it will help. (#70, nurse)

Staff acknowledged that the need to maintain patients’ confidentiality is especially important when approaching adolescents who visit the ED with a parent, as teens may not want their parents to know they are sexually active:

…if they’re here with their parents they really don’t want to do [an HIV test]. So patient confidentiality—we’ll ask their parents to step out and ask them if they want it. And most of the time they will say yes. (#73, technician)

Yet managing these concerns within the ED setting is challenging for staff and can create an uncomfortable dynamic with patients:

It’s a little difficult to separate a 13-year-old from their parent. We do it, but it’s difficult and it’s difficult for the patient then because now the parent is now asking; “Well what did they ask you? What did you do? What did you say?” Causes anxiety, so what’s the easiest thing to do? You lie. (#72, nurse)

A specific advantage of a tablet-based HIV testing intervention noted by a number of interviewees is patients’ reduced sense of judgment when being queried about sensitive topics by a computer instead of a person:

It’s a little less invasive to talk to the computer. The computer does not have any—even when we try not to, people have perceptions that somebody is going to think that maybe there’ll be a thought about their responses to the questions. So I think it’s [the tablet intervention] a good idea because it catches both—both those people who would tell a person and those people who feel more comfortable on the computer. The newer generation are computer oriented, they feel comfortable about the computer for everything now. (#72, nurse)

### Challenges Associated With Universal HIV Screening in the ED

Two additional themes not specifically anticipated by the researchers, but which emerged in staff interviews, are worthy of note: opposition, among a minority of interviewees, to the increasing number of “public health” or “primary care” services being taken on by EDs; and the difficulty of accurately identifying those ED patients who are at greatest risk for HIV and most likely to have an undiagnosed infection. More specifically, several staff spoke of the challenge presented by a subset of ED patients who are high-frequency repeat HIV testers.

Two physicians emphatically expressed their view that too many public health functions are now being asked of EDs. This again speaks to the limited availability of staff resources as a barrier to intervention implementation. Specifically, the physicians questioned whether measures such as universal HIV screening are appropriate for an ED to provide, particularly an “overburdened safety net hospital”:

You look through charts during medical reviews and the amount of unnecessary information being collected there on patients with absolutely no complaints in terms of domestic violence, in terms of everything that triage asks. And it’s—there’s click fatigue for the doctors for a lot of stuff. I think there’s fatigue in asking all these relatively useless questions. The screening for domestic violence, I think is similar to the HIV. I’m not sure that we’re doing a great deal of good to society by doing that, asking patients if they’re suicidal. (#65, physician)

In this physician’s view, the very small number of previously undiagnosed HIV infections identified by universal screening in the ED does not warrant the numerous drawbacks which include the fact that “It’s time consuming, it delays patient discharge…[and] it’s a cost that’s another unfunded mandate, I don’t know who’s paying for the tests.” However, he acknowledged that the reason “everyone wants to put more and more responsibility on the ED for primary care in the United States [is] because primary care has fallen apart.” The other physician who expressed similar doubts about public health functions as part of ED care elaborated on this point, acknowledging that many individuals in the hospital’s surrounding community have no access to healthcare apart from the ED. Moreover, he conceded, “the same patients that are very unlikely to have primary care are also more likely to use the ED as their source of primary care. And those people are more likely to have undiagnosed HIV.”

A number of interviewees commented on the subset of ED patients who are repeat testers, some of whom accept an HIV test each time it is offered. According to one physician, “So patients are getting multiple testing probably that don’t need it…it’s one thing to test high-risk patients frequently…but we have others that…just keep on getting tested and tested. I just think it’s a waste of resources.” This phenomenon presents ED staff with a conundrum because New York State requires that providers offer HIV testing to all patients aged 13–64, with limited exceptions, regardless of perceived risk. An administrator spoke of the difficulty of identifying patients at increased risk of infection so that HIV testing resources can be targeted most effectively:

There are people that have no risk factors that are just worried… and then they think, oh it’s good, a free test, I will do that. And then there’s this other end …the frequent testers who are high risk behavior people, right. So they want to test every time because between the last time they came to the ER and now they engaged in some behavior that they think they should be tested. So the influence of that is you got to take a step back and say, “Who is the person?” Right, because what you really want is keep testing the high risk behavior people because, you know, they need to be tested and there’s probably a cohort of people like me who say, “I have no risk,” who actually in fact do have risk but don’t understand that they do have risk and, you know, there needs to be some education and push on that and that I think is what your video probably does… (#78, administrator)

This administrator’s statement reflects a dilemma expressed by ED staff in various roles who acknowledged that, while frequent, repeated testing of low-risk patients strains the ED’s limited resources, they are challenged to distinguish those patients with a genuine need for frequent testing from the “worried well.” As suggested by this administrator and other staff, this problem is rooted, at least in part, in limited understanding of HIV transmission and infection dynamics among many ED patients and points to an ongoing need for HIV education.

## Discussion

In many ways, EDs present important opportunities to identify undiagnosed HIV, especially because many patients who seek care in an ED may otherwise have limited or inconsistent access to primary care, health education, and HIV testing. Our research team has developed brief (less than 10 min) technology-based interventions to facilitate HIV testing in high volume EDs, without interrupting staff workflows or impeding patient care. Although ED staff generally agree on the importance of increasing HIV test rates and agree on the potential value of a tablet-based intervention as piloted, staff interviews indicate that a lack of staff availability to administer even a largely self-directed intervention may prove a clear barrier to implementation.

Emergency department staff already face significant demands on their time. If patients are not quickly treated and promptly discharged, delays may negatively impact the care of other patients, especially at peak times. As a result, anything viewed as potentially delaying discharge, possibly including an 8- or 10-min intervention and a subsequent 20-min wait for HIV test results, may be seen as problematic. While, overall, staff interviews indicate support for increased HIV testing in the ED, a notable lack of agreement as to who should implement related interventions, and outright opposition by some physicians, raise three important questions that need to be answered before our technology can be adopted as standard practice:

1. *Who should administer the intervention?* As described in the Section “Results,” different types of ED staff (technician, nurse, and doctor) tended to suggest that staff in roles other than their own should be made responsible.

One of the doctors interviewed for the current study suggested working with students. A student-led approach was recently studied in a Baltimore ED where staff trained graduate student volunteers to test patients, increasing the number of patients tested (16). If a lack of staff availability is the greatest barrier to implementation, the inclusion of specially trained students or other volunteers could offer a potential solution, especially in academic ED settings where students are already being trained, and while testing costs are covered by public and private insurance (6). As noted earlier, our tablet-based interventions are designed to streamline the testing process and do not require skilled staff to administer; moreover, our previous research has shown that use of these interventions can consistently increase HIV test rates by roughly 30%. Thus, if tablet-based interventions such as ours were routinely administered by properly trained and supervised students or other volunteers, EDs could not only offer HIV testing to a greater number of patients, but could potentially leverage technology to do so more effectively and efficiently, with minimal disruptions to existing workflows.

2. *Are ED-based “public health” programs worthwhile?* Two physicians brought up the fact that EDs are increasingly being asked to take on public health functions such as universal HIV and HCV screening. In their view, this is problematic because the additional tasks overburden staff while the benefits gained can be difficult to gage (“low yield”). As the physicians themselves noted, much of the pressure placed on EDs to address public health issues comes from a lack of access to primary care among many of the patients most at risk for HIV, who also tend to be the patients most likely to seek care in an ED. As HIV incidence remains highest among young people and racial/ethnic minorities (17), groups who are notably less likely to have access to private medical care (18), the need for ED-based efforts to address undiagnosed HIV is likely to remain strong.
3. *How to most effectively reach ED patients with undiagnosed HIV?* A related issue raised by several interviewees concerns the difficulty in accurately identifying ED patients at highest risk for HIV and motivating them to test, as well as reaching people with undiagnosed HIV who do not report, or may not engage in, risk behaviors. This points to a continuing unmet need for widespread HIV education, particularly among young people and people of color who may be at unrecognized risk for HIV, a gap that technology-based tools may be well suited to fill.

The results of this study are subject to some limitations. Given the nature of qualitative investigations which typically involve small samples and non-probabilistic sampling methods, the results cannot be generalized to staff of large urban EDs in general or to all staff members employed by the ED that served as the study site. Because of the small sample size, it remains unclear whether interviewing more staff at the study site ED would have produced greater consensus regarding the core issues around which a notable diversity of opinion was observed (e.g., who should deliver a tablet intervention). Nonetheless, use of a purposive sampling strategy enabled the study to elicit the perspectives of a broad range of staff roles and levels of responsibility. In deference to the busy, faced-paced ED setting, interviews were intentionally brief, a design choice that maximized feasibility but necessarily limited the breadth and depth of interviews. It is possible that longer, more in-depth interviews would have elicited additional barriers to routine adoption of technology-based HIV testing interventions and/or potential solutions to these barriers.

## Conclusion

A central goal of our team’s efforts to develop, evaluate and implement effective, evidence-informed tablet interventions in ED settings is to alleviate the burden associated with delivering public health services, such as universal HIV and/or HCV screening, through the use of technology-based tools. Given the number of potentially high-risk ED patients who are still not offered HIV testing (1, 2), or who are offered HIV testing and decline (8), technology-delivered interventions in EDs offer tremendous potential to help reach those who are most at risk for HIV yet do not test. Our team’s recent projects have used qualitative interviews with participants to better understand how interventions can be made more effective/efficacious and acceptable to patients (8). A new line of research can potentially explore how intervention designs can be developed to more easily integrate into existing staff routines as well.

## Ethics Statement

This study was carried out in accordance with the recommendations of the Institutional Review Boards at Icahn School of Medicine at Mount Sinai and NDRI with written informed consent from all subjects. All subjects gave written informed consent in accordance with the Declaration of Helsinki. The protocol was approved by the Institutional Review Boards at Icahn School of Medicine at Mount Sinai and NDRI.

## Author Contributions

IA conceived of the study idea, conducted the interviews, drafted the manuscript, solicited feedback from other authors, and revised accordingly. HG and AB coded and analyzed data, then helped write up findings and revise the manuscript overall. LM, MG, and CC helped develop the study design, and each contributed to drafting and revising the manuscript at multiple points. LD provided an implementation framework used in the interviews, contributed to the design, and suggested key manuscript revisions. TB helped design the study, secured IRB permissions, helped select staff for interviews, and oversaw all data collection activities for the study.

## Conflict of Interest Statement

The authors declare that the research was conducted in the absence of any commercial or financial relationships that could be construed as a potential conflict of interest. The reviewer, DS, and handling editor declared their shared affiliation, and the handling editor states that the process nevertheless met the standards of a fair and objective review.

## Appendix A

### CFIR Interview Guide

Tell me about your experience with HIV testing in the ED.

#### External Policies and Incentives

What kind of local, state, or national performance measures, policies, regulations, or guidelines might influence the decision to implement some type of computer-based intervention?

- How could an intervention affect your organization’s ability to meet these measures, policies, regulations, or guidelines?

#### Knowledge and Beliefs about the Intervention (Part I)

1. What do you know about the intervention or its implementation?

#### Patient Needs and Resources

1. How well do you think the intervention will meet the needs of the individuals served by your organization?
  ○ In what ways will the intervention meet their needs? E.g. improved access to services? Reduced wait times? Help with self-management? Reduced travel time and expense?
2. How do you think the individuals served by your organization will respond to the intervention?
3. What barriers will the individuals served by your organization face to participating in the intervention?
4. Have you heard stories about the experiences of participants with the intervention?
  ○ Can you describe a specific story?

#### Relative Advantage

1. How does the intervention compare to other similar existing programs in your setting?
  ○ What advantages does the intervention have compared to existing programs?
  ○ What disadvantages does the intervention have compared to existing programs?
2. How does the intervention compare to other alternatives that may have been considered or that you know about?
  ○ What advantages does the intervention have compared to these other programs?
  ○ What disadvantages does the intervention have compared to these other programs?
3. Is there another intervention that people would rather implement?
  ○ Can you describe that intervention?
  ○ Why would people prefer the alternative?

#### Adaptability

1. What kinds of changes or alterations do you think you will need to make to the intervention so it will work effectively in your setting?
  ○ Do you think you will be able to make these changes? Why or why not?
2. Who will decide (or what is the process for deciding) whether changes are needed to the intervention so that it works well in your setting?
  ○ How will you know if it is appropriate to make any changes?
3. Are there components that should not be altered?
  ○ Which ones should not be altered?

#### Complexity

1. How complicated is the intervention?
  ○ Please consider the following aspects of the intervention: duration, scope, intricacy, and number of steps involved and whether the intervention reflects a clear departure from previous practices.

#### Knowledge and Beliefs about the Intervention (Part II)

1. Do you think the intervention will be effective in your setting?
  ○ Why or why not?
2. How do you feel about the intervention being used in your setting?
  ○ How do you feel about the plan to implement the intervention in your setting?
  ○ Do you have any feelings of anticipation? Stress? Enthusiasm? Why?
3. Do you think the intervention will be effective in other EDs?
  ○ Why or why not?
